# Bisphenol A Diglycidyl Ether (BADGE) and Bisphenol Analogs, but Not Bisphenol A (BPA), Activate the CatSper Ca^2+^ Channel in Human Sperm

**DOI:** 10.3389/fendo.2020.00324

**Published:** 2020-05-19

**Authors:** Anders Rehfeld, A. M. Andersson, N. E. Skakkebæk

**Affiliations:** ^1^Department of Growth and Reproduction, Copenhagen University Hospital, Rigshospitalet, Copenhagen, Denmark; ^2^International Center for Research and Research Training in Endocrine Disruption of Male Reproduction and Child Health (EDMaRC), Rigshospitalet, University of Copenhagen, Copenhagen, Denmark

**Keywords:** endocrine disruption, fertility, CatSper, male reproduction, bisphenol

## Abstract

**Aim:** Evidence suggests that bisphenol A diglycidyl ether (BADGE), bisphenol A (BPA), and BPA analogs can interfere with human male fertility. However, the effect directly on human sperm function is not known. The CatSper Ca^2+^ channel in human sperm controls important sperm functions and is necessary for normal male fertility. Environmental chemicals have been shown to activate CatSper and thereby affect Ca^2+^ signaling in human sperm. BPA has previously been investigated for effects on Ca^2+^ signaling human sperm, whereas the effects of other BPA analogs are currently unknown. The aim of this study is thus to characterize the effect of BADGE, BPA, and the eight analogs BPG, BPAF, BPC, BPB, BPBP, BPE, BPF, BPS on Ca^2+^ signaling, and CatSper in human sperm.

**Methods:** Direct effects of the bisphenols on Ca^2+^ signaling in human sperm cells were evaluated using a Ca^2+^ fluorimetric assay measuring changes in intracellular Ca^2+^. Effects via CatSper were assessed using the specific CatSper inhibitor RU1968. Effects on human sperm function was assessed using an image cytometry-based acrosome reaction assay and the modified Kremer's sperm–mucus penetration assay.

**Results:** At 10 μM the bisphenols BPG, BPAF, BPC, BADGE, BPB, and BPBP induced Ca^2+^ signals in human sperm cells, whereas BPE, BPF, BPS, and BPA had no effect. The efficacy of the chemicals at 10 μM is BPG > BPAF > BPC > BADGE > BPB > BPBP. Dose-response relations of BPG, BPAF, BPC, BADGE, BPB, and BPBP yielded EC50-values in the nM-μM range. The induced Ca^2+^ signals were almost completely abolished using the CatSper inhibitor RU1968, indicating an effect of the bisphenols on CatSper. All bisphenols, except BPBP, were found to dose-dependently inhibit progesterone-induced Ca^2+^ signals, with BPG and BPAF displaying inhibition even in low μM doses. BPG and BPAF were shown to affect human sperm function in a progesterone-like manner.

**Conclusion:** Our results show that the bisphenols BPG, BPAF, BPC, BADGE, BPB, and BPBP can affect Ca^2+^ signaling in human sperm cells through activation of CatSper. This could potentially disrupt human sperm function by interfering with normal CatSper-signaling and thus be a contributing factor in human infertility, either alone or in mixtures with other chemicals.

## Introduction

Humans are widely exposed to bisphenol A (BPA), a high-production-volume chemical (1), and bisphenol A diglycidyl ether (BADGE), both widely used in the production of, e.g., epoxy resins and food container linings (2). Due to concerns of the safety of BPA, it is increasingly substituted with analogous chemicals (3, 4). Although evidence suggests that BPA and its analogs can interfere with human male fertility (4–8), the effects directly on human sperm function are less well-studied.

Ca^2+^ signaling is a key regulator of human sperm function (9). The CatSper Ca^2+^ channel is the principal Ca^2+^ channel in human sperm (10, 11) and is activated by the female sex steroid progesterone, released in high amounts from the cumulus cells surrounding the oocyte (10, 12). The activation of CatSper by progesterone controls important sperm functions (13). A suboptimal progesterone-induced Ca^2+^ influx is associated with reduced male fertility (14–20) and men who lack functional CatSper are sterile (18, 21–29), illustrating the importance of CatSper and Ca^2+^ signaling for normal male fertility. Studies have shown that human CatSper can be promiscuously activated by various signaling molecules (30), steroids (31, 32), small molecules (33), and environmental chemicals (34–39). As only BPA, and none of its structural analogs, has previously been investigated for effects on Ca^2+^ signaling in human sperm cells (34, 40), we set out to screen BADGE, BPA, and its eight structural analogs BPG, BPAF, BPC, BPB, BPBP, BPE, BPF, BPS for effects on Ca^2+^ signaling, and CatSper in human sperm, as well as on human sperm cell function.

## Materials and Methods

### Chemicals and Reagents

Bisphenols were purchased from Sigma-Aldrich (MO, USA) and dissolved in DMSO at a stock concentration of 10 mM. Progesterone, prostaglandin-E1 (PGE1) and ionomycin were obtained from Sigma-Aldrich (MO, USA) and dissolved in DMSO at stock concentrations of 20, 20, and 1 mM, respectively. RU1968 was obtained from Professor Timo Strünker and dissolved in DMSO at a stock concentration of 10 mM. Fluo-4, AM, and BCECF, AM were purchased from Invitrogen (CA, USA). Fluorescein isothiocyanate conjugated *Pisum sativum* agglutinin (FITC-PSA), and 4,000 cP methylcellulose were obtained from Sigma-Aldrich (MO, USA). Propidium iodide (PI), Hoechst-33342 (Hoechst), and S100 were obtained from ChemoMetec A/S (Allerød, Denmark). Human serum albumin (HSA) was obtained from Irvine Scientific (CA, USA).

### Semen Samples and Ethical Approval

Healthy human volunteers donated the semen samples after their prior consent. The semen samples were produced by masturbation and ejaculated into wide-mouthed plastic containers, on the same day as the experiment and allowed to liquefy for 15–30 min at 37°C before the purification of motile sperm cells via swim-up. The volunteers were recruited from the semen donor corps, which is routinely donating samples for quality control analyses at the Department of Growth and Reproduction, Rigshospitalet. All volunteers fulfilled WHO criteria for normal sperm quality. After delivery, the samples were fully anonymized and no data on the donors fertility status, general health, or exposure to bisphenols were provided. We presumed that the donors were exposed to the same levels of bisphenols as the general population. Each donor received a fee of 500 DKK (about 75 UD dollars) per sample for their inconvenience. All samples were analyzed on the same day of delivery and destroyed immediately after the laboratory analyses. Each experimental replicate was thus based on sperm cells from a single sperm sample. Because of the full anonymization of the samples and the destruction of the samples immediately after the laboratory analyses, no ethical approval was needed for this work, according to the regional scientific ethical committee of the Capital Region of Denmark.

### Purification of Motile Sperm Cells via Swim-Up

Motile spermatozoa were isolated from the semen sample by the swim-up method (41). Briefly 1 mL of semen was gently placed in the bottom of a 50 mL tube containing 4 mL of human tubular fluid (HTF^+^) medium with the composition: 97.8 mM NaCl, 4.69 mM KCl, 0.2 mM MgSO_4_, 0.37 mM KH_2_PO_4_, 2.04 mM CaCl_2_, 0.33 mM Na-pyruvate, 21.4 mM Na-lactate, 2.78 mM glucose, 21 mM HEPES, and 4 mM NaHCO_3_, adjusted to pH 7.3–7.4 with NaOH. After 1 h at 37°C, the upper swim-up fraction was carefully removed and after two washes, the sperm concentration was determined by image cytometry (42) and the sample adjusted to 10 × 10^6^ sperm cells/ml in HTF^+^ with human serum albumin (3 mg/ml). Hereafter the sperm cells were incubated for at least 1 h at 37°C. For the experiments with capacitated sperm cells, the semen samples were resuspended in a capacitating medium with the following composition: 72.8 mM NaCl, 4.69 mM KCl, 0.2 mM MgSO_4_, 0.37 mM KH_2_PO_4_, 2.04 mM CaCl_2_, 0.33 mM Na-pyruvate, 21.4 mM Na-lactate, 2.78 mM glucose, 21 mM HEPES, and 25 mM NaHCO_3_, adjusted to pH 7.3–7.4 with NaOH. Human serum albumin (3 mg/ml) was added to the capacitating medium and the sperm cells were incubated for >3 h at 37°C in a 5% CO_2_ atmosphere.

### Measurement of Changes in [Ca^2+^]*_*i*_*

Changes in the free intracellular Ca^2+^ concentration [Ca^2+^]_*i*_ in human sperm cells were measured in 384 multi-well-plates in a fluorescence plate reader (Fluostar Omega, BMG Labtech, Germany) at 30°C as described in Rehfeld et al. (41). Briefly, sperm cells were incubated with the fluorescent Ca^2+^ indicator Fluo-4, AM (10 μM) for 45 min at 37°C. Excess dye was removed by centrifugation (700 × g, 10 min, RT) and the sperm pellet was resuspended in HTF^+^ to 5 × 10^6^ sperm cells/mL. Aliquots of 50 μL were loaded to the wells of a 384-well-plate using an automatic repeater pipette. Fluorescence was excited at 480 nm and emission was recorded at 520 nm with bottom optics. Fluorescence was recorded before and after addition of 25 μL bisphenol solutions, negative control (buffer with vehicle), positive control (progesterone, 5 μM final concentration) manually with an electronic multichannel pipette to duplicate wells. Changes in Fluo-4 fluorescence are shown as Δ*F*/*F*_0_ (%), indicating the percentage change in fluorescence (Δ*F*) with respect to the mean basal fluorescence (*F*_0_) before addition of bisphenols, positive control, and negative control. For the inhibition studies mean basal fluorescence (*F*_0_) was defined as the last 5 cycles before addition of 100 nM progesterone.

### Measurement of Changes in pH_(*i*)_

Changes in pH_(i)_ in human sperm cells were measured in 384-well-plates in a fluorescence plate reader (Fluostar Omega, BMG Labtech, Germany) at 30°C as in Schiffer et al. (34). Sperm cells were loaded with the fluorescent pH indicator BCECF (10 μM) for 15 min at 37°C. Excess dye was removed by centrifugation (700 × g, 10 min, RT) and the sperm pellet was resuspended in HTF^+^ to 5 × 10^6^ sperm/ml. Aliquots of 50 μL were loaded to the wells of the multi-well-plate. Fluorescence was excited at 440 and 480 nm (dual excitation) and emission was recorded at 520 nm with bottom optics. Fluorescence was recorded before and after addition of 25 μL of bisphenol solutions, negative control (buffer with vehicle), positive control (NH_4_CL, 30 mM final concentration) manually with an electronic multichannel pipette to duplicate wells. Changes in the ratio of BCECF fluorescence between the 440 and 480 nm excitation are shown as Δ*R*/*R*_0_ (%), indicating the percentage change in the ratio of fluorescence between the two modes of excitation (Δ*R*) with respect to the mean basal ratio of fluorescence between the two modes of excitation (*R*_0_) before addition of bisphenols, positive control, and negative control.

### Assessment of Sperm Penetration Into a Viscous Medium

Assessment of sperm penetration was done using sperm penetration tests with 4,000 cP methylcellulose (1% w/v) as an artificial viscous medium as described in Alasmari et al. (43). The viscous methylcellulose (1% w/v) medium was prepared in HTF^+^ by adding 10 mg methylcellulose per mL HTF^+^ and mixing it by rotation overnight at RT. The viscous methylcellulose (1% w/v) medium was introduced into glass capillary tubes [borosilicate microslides (VitroTubes) 0.20 mm × 2.0 mm × 10 cm (VitroCom, USA)] by capillary forces, by placing the glass tubes vertically in a 1.5 mL microfuge tube with 750 μL methylcellulose (1% w/v) for 15min. Care was taken to prevent air bubbles from entering the glass tubes. The end of the glass tube that was placed in the microfuge tube was sealed with wax (Hounisens laboratorieudstyr A/S, Denmark). Hereafter the other end was cut within the part filled with methylcellulose, just before the methylcellulose-air transition, and additional wax was added to the other end to push out a small droplet of methylcellulose at the cut end. The cut end is then placed in a 1.4 mL tube (Eppendorf, Germany) with 200 μL non-capacitated sperm sample (10 × 10^6^/ml in HTF^+^). Just prior to the insertion of the glass tubes, either bisphenols (10 μM), 5 μM progesterone (positive control), 5 μM PGE1, or 0.1% DMSO (negative control) were added to the sperm sample. The sperm cells were allowed to migrate into the methylcellulose (1% w/v) for 60 min at 37°C. The glass tube was then removed, wiped to remove residual sperm cells from the surface of the glass, placed under a UV lamp (302 nm) in a BIO-RAD Universal Hood III (BIO-RAD, CA, US) for 3 min to paralyze the sperm cells (44) and hereafter examined using phase contrast optics on an Olympus BX45 microscope at a total magnification of ×200 (Olympus, Denmark). The number of sperm cells were counted at 2 cm distance from the opening of the tube, with two fields in each of four planes counted. Throughout the study, all samples were counted by the same observer. Only experiments with a positive increment in cell density at 2 cm for the positive control compared to the negative control and with more than 40 sperm cells counted at 2 cm for the positive control were used for the analysis.

### Assessment of Acrosome Reaction

The amount of live acrosome reacted sperm cells was measured using an image cytometry-based acrosome reaction assay, as described in Rehfeld et al. (41). Briefly, capacitated sperm cells (10 × 10^6^/ml) were divided into equal aliquots and mixed thoroughly with a staining solution containing 5 μg/mL FITC-PSA, 0.5 μg/mL PI, and 10 μg/mL Hoechst in HTF^+^. Bisphenols (10 μM) were added to the aliquots of stained capacitated sperm cells together with the positive controls, ionomycin (10 μM), and progesterone (10 μM). As a negative control, HTF^+^ with 0.2% DMSO was used. After addition of bisphenols and controls the samples were mixed well and placed on a gentle mixing heating plate at 37°C. After 30 min of incubation, the aliquots were mixed thoroughly by pipetting and a 50 μL sample was drawn and added to a 100 μL aliquot of an immobilizing solution containing 0.6 M NaHCO_3_ and 0.37% (v/v) formaldehyde in distilled water. This solution was mixed well by pipetting, immediately loaded in an A2 slide (ChemoMetec A/S, Allerød, Denmark) and assessed in a NC-3000 image cytometer (ChemoMetec A/S, Allerød, Denmark). The following protocol was applied: 2-color flexicyte with Hoechst defining the sperm cells to be analyzed; Ex475-Em560/35: exposure time 3,000 ms, Ex530-Em675/75: exposure time 500 ms, with a minimum of 5,000 analyzed cells (positive for Hoechst). PI intensity as a function of FITC-PSA intensity was plotted on bi-exponential scales and specific quadrant gates were used to distinguish four groups:

1. PI positive and FITC-PSA positive cells: Acrosome reacted non-viable sperm cells.
2. PI negative and FITC-PSA positive cells: Acrosome reacted viable sperm cells.
3. PI positive and FITC-PSA negative cells: Acrosome intact non-viable sperm cells.
4. PI negative and FITC-PSA negative cells: Acrosome intact viable sperm cells.

Only experiments with an increase of live acrosome reacted sperm cells for both positive controls compared to the negative control at ≥100% were included in the analysis.

### Statistical Analysis

Data from sperm penetration tests and the acrosome reaction assay were analyzed using a mixed effects model with Geissner-Greenhouse correction. Normality was assumed based on a QQ-plot of residuals. *P*-values were corrected for multiple comparison type I error inflation by Dunnett's method. Statistical analyses were performed using GraphPad Prism 8.3.1 (GraphPad Software Inc., USA).

## Results

### Bisphenols Induce Ca^2+^ Signals in Human Sperm Cells

We investigated the 10 bisphenols BADGE, BPA, BPG, BPAF, BPC, BPB, BPBP, BPE, BPF, and BPS for their ability to induce Ca^2+^ signals in human sperm cells (Table 1), using a Ca^2+^ fluorimetric assay (34). The bisphenols were screened at a concentration of 10 μM, along a positive control (progesterone, 5 μM), and negative control (HTF^+^ with vehicle). Changes in [Ca^2+^]_*i*_ were recorded for 4 min after addition of the chemicals and controls. We calculated the relative peak Ca^2+^ signal in % induced by the bisphenols, by dividing the peak Ca^2+^ signal with that of the paired positive control, in order to compare data from the different experiments. Six of the ten bisphenols tested induced a mean relative peak Ca^2+^ signal larger than that of negative controls (HTF^+^ with vehicle) ± 3 × SD (0.0 ± 3 × 2.3%, giving a maximal value of 6.9%, Table 1). These six bisphenols were categorized as “positive hits” and investigated in further detail.

**Table 1:** Bisphenols ranked according to the mean relative peak Ca^2+^ signal induced at 10 μM, i.e., the peak Ca^2+^ signal induced by the bisphenol at 10 μM divided by the peak Ca^2+^ signal induced by progesterone at 5 μM in the same experiment.

**Rank**		**Name**	**CAS number**	**Abbrevation**	**Mean relative peak Ca^**2+**^ signal at 10 μM (in %) (*n* = 3)**	**Chemical structure**
Positive hits	1	Bisphenol G	127-54-8	BPG	109.02	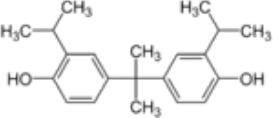
	2	Bisphenol AF	1478-61-1	BPAF	57.95	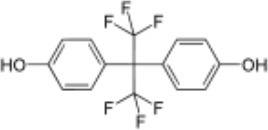
	3	Bisphenol C	79-97-0	BPC	21.67	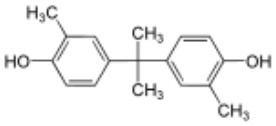
	4	Bisphenol A diglycidyl ether	1675-54-3	BADGE	14.79	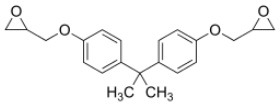
	5	Bisphenol B	77-40-7	BPB	11.75	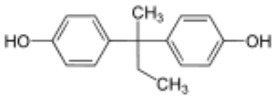
	6	Bisphenol BP	1844-01-5	BPBP	9.09	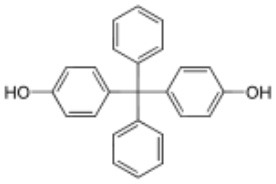
Negative hits	7	Bisphenol E	2081-08-5	BPE	5.37	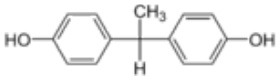
	8	Bisphenol F	620-92-8	BPF	5.09	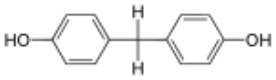
	9	Bisphenol S	80-09-1	BPS	4.41	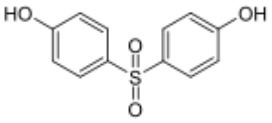
	10	Bisphenol A	80-05-7	BPA	1.44	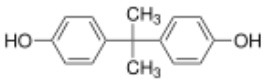

*Based on their ability to induce Ca^2+^ signals, the bisphenols are categorized into “positive hits,” which induced mean relative peak Ca^2+^ signals above that of the negative controls (HTF^+^ with vehicle) ± 3 × SD (0.0 ± 3 × 2.3 = 6.9%) and “negative hits.” CAS number, abbreviation, and chemical structure are also listed in the table*.

### Dose Response Relationship for the “Positive Hit” Bisphenols

**Figure 1:**
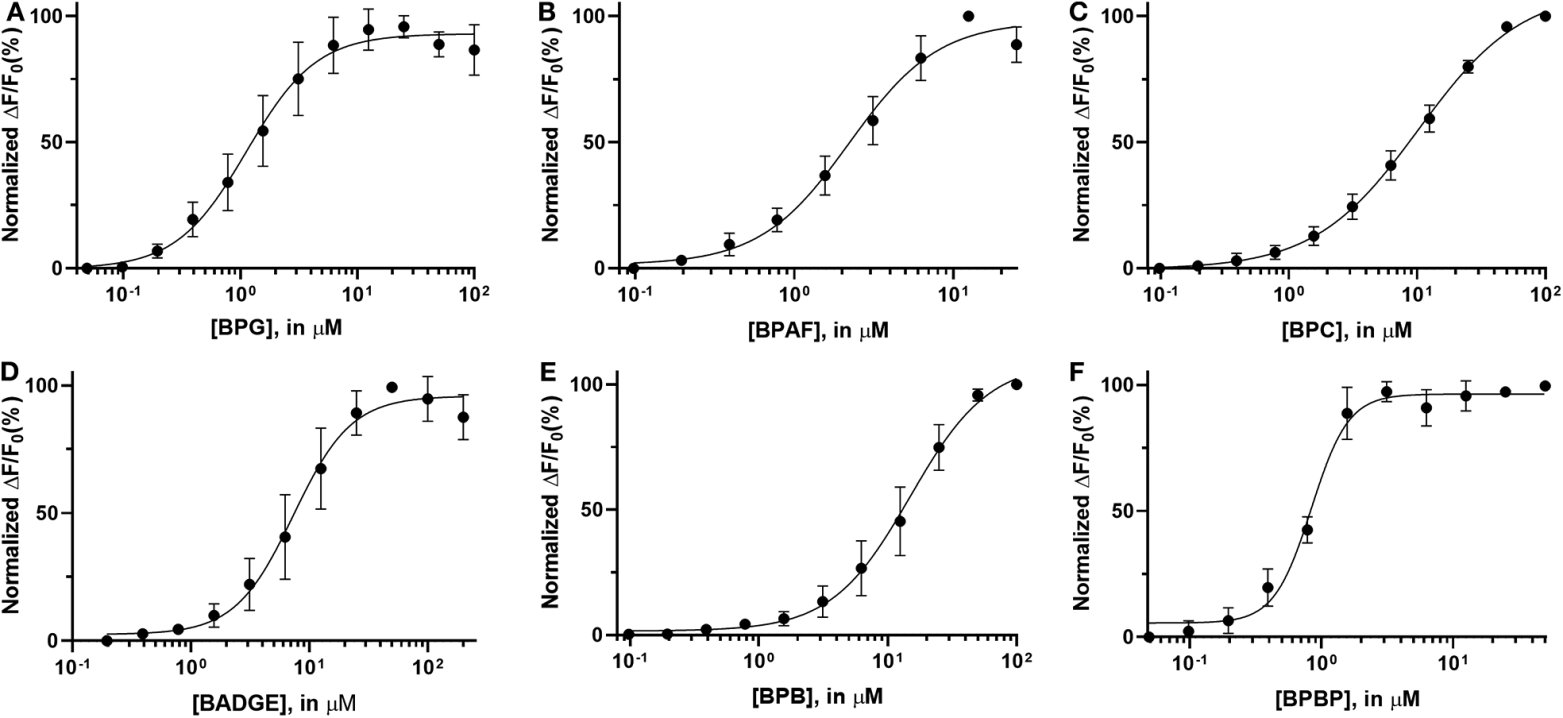
Normalized dose response curves (mean ± SD) for “positive hit” bisphenols. **(A)** BPG, **(B)** BPAF, **(C)** BPC, **(D)** BADGE, **(E)** BPB, and **(F)** BPBP, *n* = 3–7.

Dose response relations were assessed for the “positive hit” bisphenols to examine whether they induced Ca^2+^ signals in human sperm cells at physiologically relevant levels. Saturating dose response relations could be made for all six bisphenols, with mean EC_50_-values within the concentration range 0.79–15.87 μM and mean EC_05_-values within the concentration range 0.18–2.37 μM (Table 2, Figure 1).

**Table 2:** Left and middle columns: EC_50_ and EC_05_ for the dose response curves (mean and SD, *n* = 3–7) of all “positive hit” bisphenols.

	**EC**_****50****_, **μM**		**EC**_****05****_, **μM**		**IC**_****50****_, **μM**	
	**Mean**	**SD**	**Mean**	**SD**	**Mean**	**SD**
BPG	1.27	0.61	0.18	0.16	1.86	0.80
BPAF	2.40	0.93	0.36	0.09	12.3	4.12
BPC	10.26	1.83	0.70	0.27	45.2	4.25
BADGE	8.18	3.88	1.82	1.01	–	–
BPB	14.87	4.42	2.37	1.75	39.9	8.05
BPBP	0.79	0.06	0.38	0.26	–	–

*Right column: IC_50_ (mean and SD, n = 3–5) for the dose response curves of Ca^2+^ signals induced by 100 nM of progesterone after pre-incubation of human sperm cells with various concentrations of the 6 “positive hit” bisphenols. Note that the dose response data generated from the preincubation experiments with BADGE and BPBP could not be used to estimate IC_50_-values*.

### Bisphenols Induce Ca^2+^ Signals Through an Activation of CatSper

**Figure 2:**
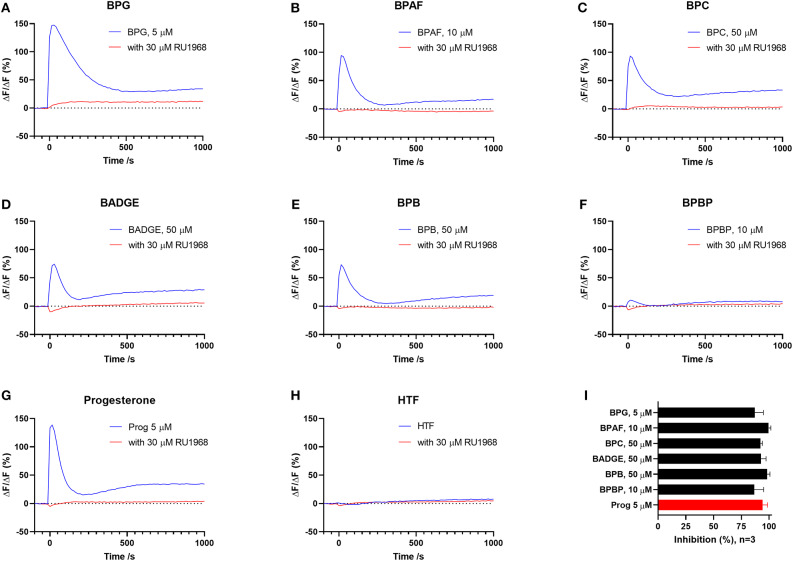
Ca^2+^ signals induced by **(A)** BPG, **(B)** BPAF, **(C)** BPC, **(D)** BADGE, **(E)** BPB, **(F)** BPBP, **(G)** the endogenous CatSper ligand progesterone, and **(H)** HTF^+^ buffer in the absence and presence of CatSper inhibitor RU1968, 30 μM. **(I)** Mean inhibition (in %) of the induced Ca^2+^signal ±SD in the presence of RU1968, 30 μM (*n* = 3).

**Figure 3:**
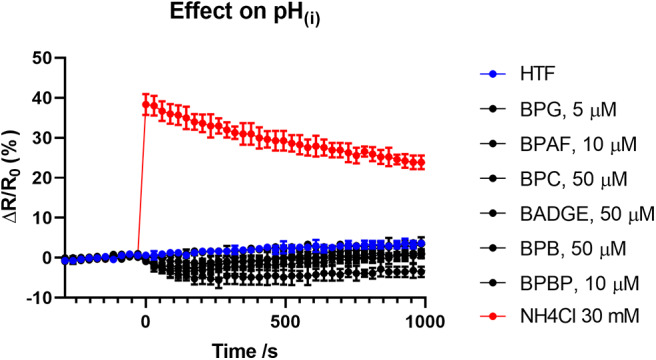
Changes (mean ± SD) in pH_(i)_ induced by the bisphenols (5–50 μM), HTF^+^ buffer and positive control NH_4_Cl at 30 mM (*n* = 3).

To test if the six “positive hit” bisphenols induced Ca^2+^ signals through CatSper, we used the novel and specific CatSper inhibitor RU1968 (13). We compared the Ca^2+^ signals induced by the bisphenols at doses inducing peak Ca^2+^ signals (5–50 μM) and progesterone at 5 μM, in the presence or absence of 30 μM of RU1968 (Figure 2). We found that the Ca^2+^ signals induced by all six bisphenols, like that of progesterone, were highly inhibited by RU1968. This strongly indicates that the bisphenols induce Ca^2+^ signals via a specific activation of CatSper in human sperm cells. Furthermore, the shape of the Ca^2+^ signals induced by the bisphenols at these doses, except BPBP, which only induce a small Ca^2+^ signal, resembles that induced by progesterone (Figure 2). This further indicates an action of the bisphenols on CatSper. As CatSper can be activated both by the endogenous ligands progesterone and prostaglandins, as well as by intracellular alkalization, we examined if the bisphenols induced changes in pH_(i)_. At bisphenol doses inducing peak Ca^2+^ signals (5–50 μM) no increase pH_(i)_ was observed (*n* = 3, Figure 3). This suggests that the bisphenols most likely act on the ligand-dependent pathway of either progesterone or prostaglandins leading to activation of CatSper.

### Bisphenols Dose-Dependently Inhibit Progesterone-Induced Ca^2+^ Signals

**Figure 4:**
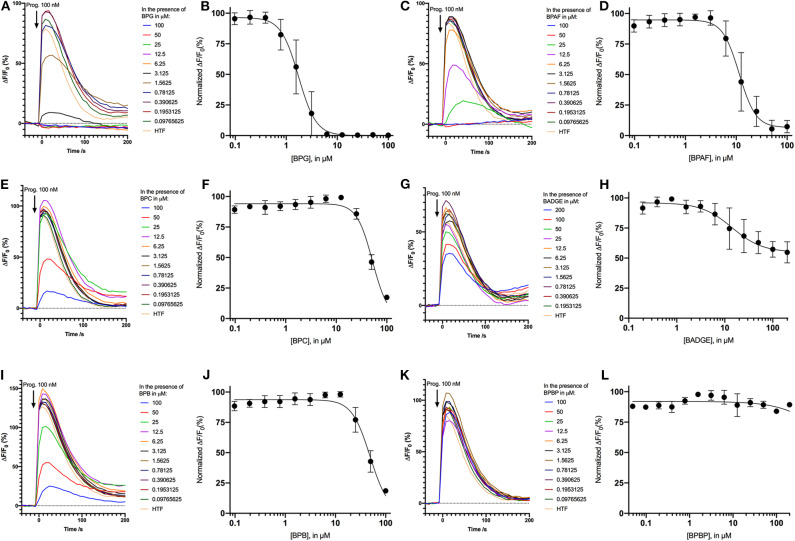
Inhibition of Ca^2+^ signals induced by 100 nM progesterone after 5 min of pre-incubation with the negative control HTF and different concentrations the bisphenols: **(A)** BPG, **(C)** BPAF, **(E)** BPC, **(G)** BADGE, **(I)** BPB, and **(K)** BPBP. Normalized dose response relations (*n* = 3−5) of Ca^2+^ signals induced by 100 nM progesterone after 5 min of pre-incubation with the different concentrations the bisphenols: **(B)** BPG, **(D)** BPAF, **(F)** BPC, **(H)** BADGE, **(J)** BPB, and **(L)** BPBP.

As the bisphenols were found to induce Ca^2+^ signals through CatSper we examined whether pre-incubating the human sperm cells with the bisphenols could inhibit progesterone-induced Ca^2+^ signals. We compared the amplitude of the Ca^2+^ signals induced by 100 nM of progesterone in human sperm cells after 5 min of pre-incubation with serially diluted doses of the bisphenols or a negative buffer control. Our results showed that all bisphenols, except BPBP, were able to dose dependently inhibit the progesterone-induced Ca^2+^ signals (Figure 4). The mean IC_50_-values estimated from the fitted dose response curves were within the concentration range 1.86–45.2 μM (Table 2).

### Effects of Bisphenols on CatSper-Mediated Human Sperm Responses

**Figure 5:**
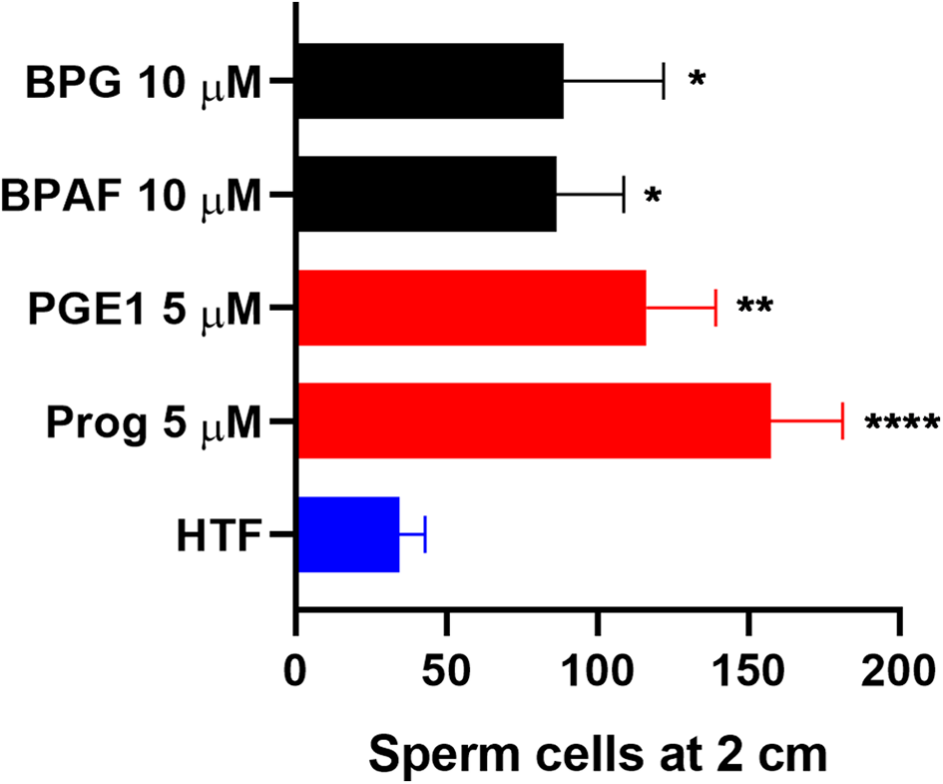
Human sperm cells at 2 cm into a viscous medium (mean ± SEM) after treatment with negative control (HTF^+^ with 0.1% DMSO “HTF”), positive controls (5 μM progesterone “Prog” and prostaglandin E1 “PGE1”), 10 μM BPG, and 10 μM BPAF (*n* ≥ 5). Statistics from multiple comparison between negative control and treatments: ****adjusted *P* ≤ 0.0001; **adjusted *P* = 0.0029; *adjusted *P* ≤ 0.0295.

**Figure 6:**
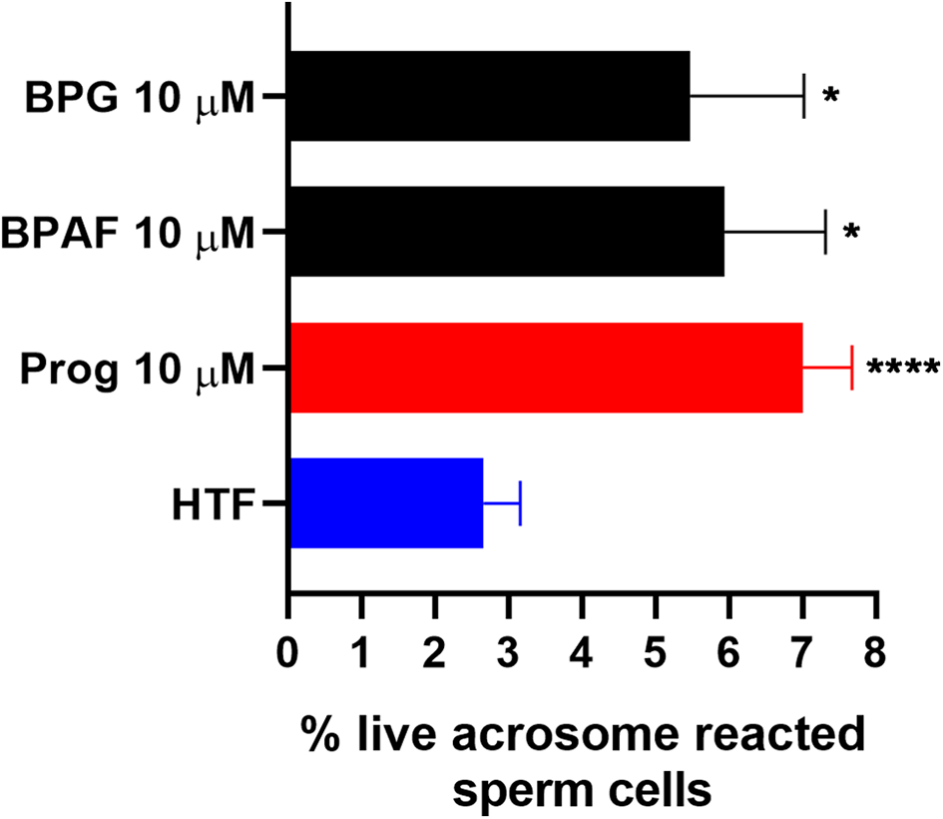
Percentage live acrosome reacted sperm cells (mean ± SEM) after 30 min treatment of capacitated human sperm cells with negative control (HTF^+^ with 0.2% DMSO “HTF”), positive control (10 μM progesterone “Prog”), 10 μM BPG, and 10 μM BPAF (*n* ≥ 8). Statistics from multiple comparison between negative control and treatments: ****adjusted *P* ≤ 0.0001; *adjusted *P* ≤ 0.0249.

To examine whether the bisphenols could affect CatSper-mediated human sperm responses, we examined the effect of the two most efficacious bisphenols, BPG and BPAF at 10 μM, on sperm penetration into a viscous medium, as well as on acrosome reaction. BPG and BPAF were found to induce a significant increase in the numbers of human sperm cells penetrating into a viscous medium (Figure 5), similar to the effect of the endogenous CatSper ligands, progesterone, and prostaglandin E1 at 5 μM. Furthermore, BPG and BPAF were found to induce a significant increase in live acrosome reacted sperm cells (Figure 6), similar to the effect of the endogenous CatSper ligand progesterone at 10 μM, in capacitated human sperm cells.

## Discussion

Our study showed that BADGE and the five bisphenol analogs BPG, BPAF, BPC, BPB, and BPBP can induce Ca^2+^ signals in human sperm cells at 10 μM, whereas BPA and three other bisphenols BPE, BPF, and BPS induced no Ca^2+^ signals in human sperm cells at this concentration (Table 1). The efficacy of the chemicals at 10 μM was BPG > BPAF > BPC > BADGE > BPB. It seems that the bisphenols with larger/bulkier side chains are more efficacious and that relatively small molecular differences between the bisphenols can alter their effects significantly (Table 1). This is in line with a previous study showing that the read-across approach was non-applicable for otherwise structurally comparable bisphenols (45). Interestingly, low doses of BPAF, BPB, BPF, BPS, and BPA have all been shown to induce Ca^2+^ signals in SKBR3 cells via the G protein-coupled estrogen receptor (GPER) (46), with BPAF and BPB being more efficacious than BPF, BPS, and BPA. This is somewhat similar to our findings, although we in our assay see no effect for BPF, BPS, and BPA. Furthermore, even though BPA showed no effect in our assay, it has been shown both to activate (47) and inhibit other voltage-activated Ca^2+^ channels (48).

The induced Ca^2+^ signals could be used to form saturating dose response curves for all six “positive hit” bisphenols (Figure 1). The EC_50_-values estimated from these curves ranged from 0.79 to 14.87 μM and the lowest effective dose values (EC_05_) ranged from 0.18 to 2.37 μM (Table 2). In the literature, we could only identify human plasma or serum levels for BPAF, BADGE, and BPB out of the six “positive hit” bisphenols (49–53). A reported maximal human serum concentration of BADGE (3.45 μM) (50) is above the EC_05_ estimated in our study (1.82 μM), whereas the reported maximal human serum levels of BPAF (0.05 μM) (50) and BPB (0.59 μM) (50) are below the estimated EC_05_-values of 0.36 μM for BPAF and 2.37 μM for BPB.

We found that the induced Ca^2+^ signals were almost completely inhibited by the specific CatSper inhibitor RU1968 (Figure 2), like the Ca^2+^ signal induced by the endogenous CatSper ligand progesterone. This indicates that the six bisphenols induce Ca^2+^ signals in human sperm cells via CatSper. Furthermore, the shape of the Ca^2+^ signals induced by all bisphenols, except BPBP, which only induced a small peak Ca^2+^ signal, resembled that of the Ca^2+^ signal induced by progesterone (Figure 2), similarly suggesting an effect of these bisphenols on CatSper. Human CatSper can be activated by a ligand-dependent pathway, by the endogenous CatSper ligands progesterone and prostaglandins (10, 12), as well as by a ligand-independent pathway through intracellular alkalization (10, 12). Our data showed that the induction of Ca^2+^ signals by the bisphenols is not due to an increase in pH_(i)_ (Figure 3), suggesting that the bisphenols act on the ligand-dependent pathways of either progesterone or prostaglandins leading to activation of CatSper in human sperm cells (10, 12). Interestingly, progesterone has been suggested to activate CatSper through an activation of the enzyme ABHD2, whereas the molecular target of prostaglandins leading to CatSper activation remains unknown (54). The direct action of the bisphenols on the ligand-dependent pathway leading to activation of CatSper in human sperm cells, is similar to what has been shown for multiple other environmental chemicals previously (34, 35, 55).

Pre-incubation of the human sperm cells with the bisphenols BPG, BPAF, BPC, BADGE, and BPB was found to dose-dependently inhibit progesterone-induced Ca^2+^ signals (Figure 4). BPG and BPAF were found to be much more potent inhibitors of progesterone-induced Ca^2+^ signals than the other bisphenols, which only inhibited progesterone-induced Ca^2+^ signals at high μM doses (Table 2). Exposure of human sperm cells to these bisphenols may thereby inhibit the action of progesterone on CatSper, as has been shown for other environmental chemicals acting on the ligand-dependent pathway (34–36, 38).

In addition, our results showed that the two most efficacious bisphenols at 10 μM, BPG and BPAF, could both increase sperm penetration into a viscous medium, like the response induced by the endogenous CatSper ligands progesterone and PGE1 (Figure 5), and induce acrosome reaction in capacitated human sperm cells, similar to the response induced by progesterone (Figure 6). Again, this is in line with previous studies where other environmental chemicals activating CatSper were found to exert progesterone-like effects on human sperm function (34, 36–38, 56).

Only few studies have examined the effect of bisphenols on human sperm cell function. One study showed that very high doses of BPA (≥300 μM) induced mitochondrial dysfunction in human sperm (57), another study showed that BPA at 0.1 nM−1 μM could affect human sperm motility parameters and that BPA at 1 μM could induce a rapid, transient increase in [Ca^2+^]_*i*_ in a whole population of observed single human sperm cells (40), whereas BPA at 0.1, 1, and 10 μM did not affect [Ca^2+^]_*i*_ in human sperm cells in a large screening of environmental chemicals by Schiffer et al. (34). Our results here support the findings by Schiffer et al. (34) that BPA at concentrations up to 10 μM do not induce Ca^2+^ signals in human sperm cells.

Our findings add BADGE and the five bisphenol analogs BPG, BPAF, BPC, BPB, and BPBP to the growing list of environmental chemicals that can induce Ca^2+^ signals in human sperm cells through CatSper (34–39). Studies have shown that chemicals acting on CatSper can cooperate in low dose mixtures to activate CatSper both additively (34, 35) and synergistically (55). As humans in the industrialized part of the world are suggested to be exposed to thousands of environmental chemicals (58), such a low dose mixture exposure scenario is quite realistic. This indicates that the bisphenols could affect Ca^2+^ signaling in human sperm cells even at doses well below the EC_05_, when present in mixtures with other chemicals acting on CatSper. This is important as only BADGE has been found with a maximal serum concentration (3.45 μM) (50) above the EC_05_ estimated in our study (1.82 μM).

Whether exposure of the human sperm cells, either within the male or female reproductive tract, to environmental chemicals acting on CatSper can interfere with the fertilization process remains to be shown. However, the fact that impaired progesterone-signaling is associated with reduced male fertility (14–20) and that men who lack functional CatSper are completely infertile (18, 21–29) hints that environmental chemicals interfering with this signaling pathway could make it more difficult for the human sperm cells to successfully locate and fertilize the oocyte. As our experiments have been performed on sperm cells *in vitro* future studies would be needed to validate our results and to examine the effects of exposure to bisphenols on fertility *in vivo*.

## Data Availability Statement

The raw data supporting the conclusions of this article will be made available by the authors, without undue reservation, to any qualified researcher upon request.

## Ethics Statement

Because of the full anonymization of the samples and the destruction of the samples immediately after the laboratory analyses, no ethical approval was needed for this work, according to the regional scientific ethical committee of the Capital Region of Denmark. The patients/participants provided their written informed consent to participate in this study.

## Author Contributions

AR, AA, and NS conceived the study and drafted the manuscript. AR designed, planned, and performed the experiments.

## Conflict of Interest

The authors declare that the research was conducted in the absence of any commercial or financial relationships that could be construed as a potential conflict of interest.
